# Mental health-related knowledge, attitudes and behaviours in a cross-sectional sample of australian university students: a comparison of domestic and international students

**DOI:** 10.1186/s12889-023-15123-x

**Published:** 2023-01-25

**Authors:** Anthony D. LaMontagne, Clare Shann, Erin Lolicato, Danielle Newton, Patrick J Owen, Adrian J. Tomyn, Nicola J. Reavley

**Affiliations:** 1 Institute for Health Transformation & School of Health & Social Development, Deakin University, Geelong, VIC Australia; 2 Shann Advisory, Melbourne, VIC Australia; 3 Diversity & Inclusion, Deakin University, Geelong, VIC Australia; 4 Melbourne School of Population & Global Health, University of Melbourne, Melbourne, VIC Australia; 5 Institute for Physical Activity and Nutrition (IPAN), School of Exercise and Nutrition Sciences, Deakin University, Geelong, VIC Australia; 6 School of Psychology, Deakin University, Melbourne, VIC Australia; 7 Bupa Asia Pacific, Adelaide, SA Australia

**Keywords:** Mental health, Mental health literacy, Stigma, Help-seeking, University, Tertiary, Student, Mental health policy

## Abstract

**Background:**

There are growing concerns about the mental health of university students in Australia and internationally, with universities, governments and other stakeholders actively developing new policies and practices. Previous research suggests that many students experience poor mental health while at university, and that the risk may be heightened for international students. Mental health-related knowledge, attitudes and behaviours are modifiable determinants of mental health and thus suitable targets for intervention. This study assessed the mental health-related knowledge, stigmatising attitudes, helping behaviours, and self-reported experiences of mental health problems in the student population of a large multi-campus Australian university, and conducted a comparative assessment of international and domestic students.

**Methods:**

Participants were 883 international and 2,852 domestic students (overall response rate 7.1%) who completed an anonymous voluntary online survey that was sent to all enrolled students in July 2019 (n = ~ 52,341). Various measures of mental health-related knowledge, attitudes and helping behaviours were assessed. A comparative analysis of international and domestic students was conducted, including adjustment for age and sex.

**Results:**

Overall, there was evidence of improvements in mental health-related knowledge, attitudes and behaviours relative to previous studies, including higher depression recognition, intentions to seek help, and reported help-seeking behaviour. Comparative analysis indicated that international students scored predominantly lower on a range of indicators (e.g., depression recognition, awareness of evidence-based forms of help); however, differences were narrower difference between the two groups compared to what has been reported previously. Finally, some indicators were more favourable among international students, such as higher help-seeking intentions, and lower prevalence of self-reported mental health problems compared to domestic students.

**Conclusion:**

Though there were some important differences between domestic and international students in this study, differences were narrower than observed in previous studies. Study findings are informing the on-going implementation and refinement of this university’s student mental health strategy, and may be used to inform evolving policy and practice in the university sector.

**Supplementary Information:**

The online version contains supplementary material available at 10.1186/s12889-023-15123-x.

## Background

The mental health and wellbeing of university or tertiary students is a core concern for universities around the world. There have been several Australian and international studies suggesting that a high proportion of students experience poor mental health while at university [1–9]. This includes the World Mental Health Surveys’ International College Student Initiative, a large-scale WHO collaborative survey of 19 colleges/universities in eight countries [8, 10], undertaken to address “rising rates of mental disorders” (Auerbach, 2018, page 624 [8]) among university students. High rates of poor mental health has a profound impact on academic outcomes [10]—whether due to life stage (with 75% of mental disorders having their first onset before age 24 [11]) or whether elevated relative to non-student peers. This is of growing concern to universities in Australia and internationally, with a focus on understanding the extent and nature of the issues so that appropriate and accessible prevention, promotion and support programs can be put in place [9, 12].

In Australia, some studies of university students suggest a high prevalence of mental health problems or disorders compared to general adult populations, whereas others do not. For example, in a 2016 nation-wide survey of students in 40 Australian universities and 30 technical and further education institutes, undertaken by the National Union of Students and Headspace in 2016, 67% of students aged 16 to 25 years self-rated their mental health as “fair” or “poor”, and a substantial proportion (24%) of those who had accessed on-campus counselling services rated their experience as negative [13]. Studies with stronger designs such as large-scale population surveys accounting for age or life stage, however, suggest little difference between students and their non-student peers with respect to high levels of psychological distress, though they have shown higher levels of moderate distress [14].

Turning to the mental health of international students in Australia, there is some evidence of a particular concern for this group. A 2017 report by a large Australian youth mental health service provider [15] identified international students as a cohort of young people in Australia who may be at higher risk of poor mental health, due to factors such as adjusting to new environments, disconnection from family and friends, different cultural constructs of mental health problems, experiences of racism, lapsing private health insurance resulting in limited access to mental health services, and work-study conflict arising from financial pressures [14, 15]. More recently, a 2020 (Australian) Productivity Commission Inquiry Report on Mental Health highlighted particular concerns and needs of international students in Australia, including potentially elevated levels of distress and other mental health problems; as well as elevated risk of suicidal behaviours, and barriers to access to mental health services [9]. The Productivity Commission’s recommendations included expanding online mental health services, improving health insurance cover for international students for mental health services, and requiring all tertiary institutions to have a student mental health and wellbeing strategy. The latter recommendation is complemented and informed by the 2020 Australian University Mental Health Framework [12], which aims to provide nationally consistent guidance for universities to support student mental health and wellbeing.

University and other strategies to improve population mental health cover a wide spectrum of prevention, treatment and maintenance [16]. In this paper, we consider the role of mental health-related knowledge, attitudes and behaviours as modifiable attributes for prevention and promotion. Mental health literacy has been defined as the “knowledge and beliefs about mental disorders which aid their recognition, management or prevention” [17]. Mental health literacy entails seven domains: the ability to recognise specific disorders; knowing how to seek mental health information; knowledge of risk factors and causes; knowledge of self-treatments; knowledge of professional help available; and attitudes that promote recognition and appropriate help-seeking. High mental health literacy has been shown to be related to greater intentions to seek help, including among university student populations [18]; conversely, poor mental health literacy reduces the likelihood of seeking appropriate professional treatments and therapies for mental health problems [19, 20]. Stigmatising attitudes can be a barrier to service use and may also lead to unsupportive, discriminatory behaviours towards people with mental health problems [21, 22]. Stigmatising attitudes (sometimes considered part of mental health literacy) and help-offering and help-seeking behaviours are important complements to mental health literacy. Interventions on mental health literacy, stigma reduction, and helping behaviours are often combined [23–25], and such interventions have also been employed as a strategy to reduce mental health disparities between groups [26].

Mental health-related knowledge, attitudes and behaviours in students have been investigated in a number of Australian studies of domestic and international university students. For example, in a study involving telephone interviews with 774 students, Reavley et al. found that while recognition of mental health problems was relatively high, intentions to seek help from services were low [27]. In the same study, several factors associated with higher levels of stigmatising attitudes were identified, including being male, of younger age, lower education levels, being born outside of Australia and lack of recognition of depression symptomology [27]. Clough et al. conducted an online survey of 209 international and 148 domestic students from an Australian university and associated tertiary college [28]. International students reported lower levels of mental health literacy, help-seeking attitudes and help-seeking intentions for suicidal ideation than domestic students. However, the researchers found no difference between the two student groups for help-seeking intentions for emotional problems or psychological distress [28]. Similar findings were made in a study of Chinese-speaking international students attending an Australian university [3]. Hence, it appears that the need for tailored mental health strategies for international versus domestic students may vary by institution or other factors.

The current paper is part of a larger project. The project was developed in the context of a single, large university responding to the need for more coordinated and comprehensive action on student mental health. Based on consultation with a range of stakeholders (including the authors) and a review of its offerings in terms of mental health promotion, student supports, and clinical services, Deakin University first launched a Student Mental Health Strategy in 2019 [29]. The Strategy was revised in 2022 to align with the six principles expressed in the 2020 Australian University Mental Health Framework [12], and has been published in a new Strategy for the period 2023–2025 [29]. From the university partner perspective, the purpose of the research was to provide granular needs assessment information for action to guide the on-going evolution of the university’s mental health programs, and to contribute to baseline assessment for future evaluation of the effectiveness of the university’s Mental Health & Wellbeing Strategy. The project was initiated in 2018 and—in an attempt to optimise the relevance and utility of the research for application to policy and practice—was conducted as a collaboration between researchers, relevant professional staff at the university, and a subject matter expert employed by the funder.

The current paper reports on one element of the larger project. The specific aims of the current paper were to:

1) Assess the current state of mental health-related knowledge, attitudes and behaviours among students in a large multi-campus Australian university; and.

2) Conduct a comparative analysis of mental health-related knowledge, attitudes and behaviours between international and domestic students in the same large multi-campus Australian university.

## Methods

### Study design & population

A cross-sectional online survey was conducted in July of 2019. The entire student population of a large Australian university (including four geographically distributed physical campuses plus on-line ‘Cloud-based’ students) was directly emailed a link to the voluntary survey titled ‘*Inclusion and Wellbeing Survey’* from the Dean of Students. The survey protocol was reviewed and approved by the University Human Research Ethics Committee. The total student population, inclusive of all undergraduate and postgraduate, part-time and full-time, domestic and international, physical campus and Cloud-based students in Trimester 1 of 2019, was 52,341.

The survey was completed by students at a time of their own choosing and under conditions of privacy and anonymity. On completion, students were invited to enter a prize draw to win a $100 gift card and/or to volunteer to participate in a qualitative follow-up study (decoupled from survey response data). After excluding responses with less than 40% completed items (590 cases), 3,735 responses were received for an effective response rate of 7.1% (3,735/52,341). Census sampling and a broad survey emphasis on social inclusion and wellbeing were employed in an attempt to minimise the potential for selection bias. Student characteristics in the obtained sample were compared to university student enrolment data (see Appendix A).

### Measures

*Demographics.* Demographic characteristics were obtained, including age, gender, international or domestic student status, country of birth and current degree program.

*Depression recognition.* Depression recognition was measured using a short vignette of a fictional, non-gendered 21-year-old person called ‘Sammi’. The vignette outlined various symptoms that meet the diagnostic criteria (in both the Diagnostic and Statistical Manual for Mental Disorders, 4th edition; and the International Classification of Disorders, 10th edition) for major depression [30, 31]. Respondents were then asked ‘What, if anything do you think is wrong with Sammi?*’* and presented with a list of sixteen choices. This measure (see Appendix B) was adapted from a similar measure which has been used in multiple studies [27, 32, 33].

*Help-seeking intentions.* All respondents were asked if they had a problem right now like Sammi, would they seek help. Response options were: yes, no or don’t know.

*Intended sources of help*. Respondents who responded ‘yes’ to the ‘Help-seeking intentions’ item were asked where they would go for help and were presented with a list of 18 sources of help, categorised into groups a) university services b) other sources of professional help and c) informal sources of help.

*Beliefs about usefulness of help*. Respondents were presented with a list of 13 interventions that might be helpful to Sammi, as judged by 66% or more of surveyed health professionals rating an intervention as helpful or harmful in an Australian sample of psychologists, psychiatrists and general practitioners (including, for example, specific therapies and medications, physical exercise, information seeking, using marijuana to relax and joining support groups [32, 34]). They were asked to rate each as either ‘helpful’, ‘harmful’, ‘neither’ or ‘unsure’. This question set was summarised as the percentage of the 13 interventions in the list correctly identified as helpful according to current medical opinion.

*Personal stigma*. The Depression Stigma Scale [35] was used to assess stigmatising attitudes towards Sammi. Responses to seven items were recorded on a 5-point Likert scale (1 = strongly disagree; 5 = strongly agree). Two subscales: ‘weak not sick’ (four items about beliefs that the person was weak, not ill, could control their behaviour, and should be avoided, scored 0–4) and ‘dangerous and unpredictable’ (three items about dangerousness and unpredictability, scored 0–3), were computed. Responses of agreed or strongly agreed were coded as 1 point, and the items were summed. Finally, there was a single item measure on whether they would tell anyone if they had a problem like Sammi’s. Higher values represent higher stigma.

*Social distance*. The Social Distance scale [36] was used to assess willingness to have social contact with the person described in the depression vignette, which we consider to be another measure of stigmatising attitudes. The scale includes five items, for example: ‘Would you be happy to go out with Sammi on the weekend?’, with response options on a 5-point Likert scale (Yes, definitely = 1; Yes, probably = 2; Unsure = 3; Probably not = 4; Definitely not = 5). The social distance measure items are summed to get the total social distance score, with higher scores indicating preference for higher social distance or higher stigma.

*Self-reported mental health problems*. Respondents were presented with a definition of a mental health problem (“A mental health problem is a cluster of symptoms that affects a person’s thinking, emotional state and behaviour, and disrupts the person’s ability to work or carry out other daily activities and engage in satisfying personal relationships. The problem lasts for a period of weeks or more” (see Appendix C), and were asked ‘in the last 12 months, have you had a mental health problem?’ Response options were yes, no or prefer not to say. This measure was created to aid interpretation of responses to questions on help-seeking.

*Experience of help-seeking.* Respondents who reported yes to self-reported mental health problem(s) were asked if they had sought help and, if so, where did they seek help. They were then presented with a list of sources for help, categorised into university services, other sources of professional help and informal sources of help. Those respondents who indicated they had a mental health problem in the last 12 months but did not seek help for it were asked ‘Did you try to deal with the problem on your own?’

*Mental health problems of close contacts and help-offering.* Respondents were asked if anyone in their family or close circle of friends had a mental health problem in the last 12 months. Response options were yes, no, don’t know or prefer not to say. If they responded affirmatively, they were asked ‘did you do anything to help this person?’ Again, if they responded ‘yes’, they were asked ‘what did you do?’ and were provided with 6 response options reflecting various types of help. A measure of help-offering was included to inform the university’s consideration of the promotion of help-offering as part of the university’s mental health promotion programs.

*Confidentiality of health services:* An interview study with students and professional staff at the university was conducted prior to and to inform the design of the quantitative survey reported on in this paper [37, 38]. University professional staff highlighted the possibility that there would be differences in understanding of the confidentiality of health services between international and domestic students, noting concerns about this as a potential barrier to help-seeking. The authors composed two survey items to assess awareness of confidentiality of university and community-based services, preceded by a plain language definition (full text of items provided in Appendix D).

### Analysis

Summary measures were computed as appropriate to the measure (e.g., frequencies for categorical, means for numeric). Comparisons were made between domestic and international students on various measures and were tested for statistical significance by conducting chi-square (categorical) or t-tests (continuous), with two-sided p-values reported. We also assessed differences between domestic and international students using multiple logistic regression with adjustment for age and gender. All statistical analyses were conducted in Stata 16.0 (Stata Corp, College Station TX, USA).

## Results

The sample was broadly representative of the student body on key demographic characteristics, as determined by comparison with census/enrolment data (see Appendix A). The sample was not statistically significantly different from the student population with regards to socio-economic status, indigeneity, campus location (including cloud/online), and international versus domestic student status. Though the sample matched the University’s enrolled student data, with 24% international students, the sample differed from the population with respect to regional representation, mainly due to under-representation of students from China (26% of international student population, yet only 10% in sample, Appendix A). Further, female and older students and students with a disability were over-represented (though the two data sources used different definitions to describe presence of disability).

Demographic and other characteristics of the sample are presented in Table 1. International students had comparable gender distribution to domestic students. International students also had an older age profile than domestic students, yet were more likely to be enrolled in undergraduate study. Table 1 details country of birth in relation to domestic/international student status. Of note, 65% of respondents were Australian-born, yet 75% identify as domestic students because domestic students can be born overseas (e.g., born to Australian parent while overseas, or if are born elsewhere but become Australian citizens). For example, over 90% of the 60 UK-born survey respondents were ‘domestic’ students.

**Table 1:** Descriptive Statistics for Demographic Characteristics of International Students, Domestic Students and the Total Sample

	International (*n* = 883) *n* (%)	Domestic (*n* = 2,852) *n* (%)	Total (*n* = 3,735) *n* (%)
Gender			
Man	449 (50.8)	648 (22.7)	1097 (29.4)
Woman	422 (47.8)	2140 (75.0)	2562 (68.6)
Other (gender diverse, prefer not to say/self-describe)	12 (1.4)	64 (2.2)	76 (2.0)
Age range			
18–20 years	144 (16.3)	764 (26.8)	908 (24.3)
21–24 years	324 (36.7)	699 (24.5)	1023 (27.4)
25–29 years	289 (32.7)	465 (16.3)	754 (20.2)
30–34 years	86 (9.7)	275 (9.6)	361 (9.7)
35 + years	40 (4.5)	649 (22.8)	689 (18.5)
Country of birth			
Australia	4 (0.4)	2,438 (85.5)	2,442 (65.4)
China	88 (10.1)	13 (0.5)	101 (2.7)
India	329 (37.2)	33 (1.2)	362 (9.7)
New Zealand Sri Lanka United Kingdom Philippines Other (< 50 count)	0 (0) 65 (7.4) 5 (0.6) 58 (6.7) 334 (37.8)	51 (1.8)) 14 (0.5) 55 (1.9) 21 (0.7) 227 (7.9)	51 (1.4) 79 (2.1) 60 (1.6) 79 (2.1) 561 (15.0)
Current level of study			
3-year undergraduate degree	207 (23.4)	1131 (39.7)	1338 (35.8)
4 + year undergraduate degree	53 (6.0)	588 (20.1)	641 (17.2)
Double degree	316 (11.1)	18 (2.0)	334 (8.9)
Postgraduate degree by coursework	422 (47.8)	544 (19.1)	966 (25.9)
Postgraduate degree by research	103 (11.7)	169 (5.9)	272 (7.3)
Other diploma/certificate/degree	80 (9.1)	104 (3.6)	184 (4.9)

*Depression recognition*. Most respondents correctly identified depression (83%, n = 1,791) in the vignette. International students were significantly less likely than domestic students to identify depression (86% versus 74%), including after adjusting for age and sex (Table 2). With a more inclusive set of correct answers (e.g., depression, mental illness, psychological/mental/emotional problem), the vast majority of respondents correctly identified at least one mental health problem (94%, n = 1,791), with no meaningful difference between domestic and international students (Table 2).

**Table 2:** Mental health literacy measures in student sample overall (total), domestic and international students in bivariate analyses, and domestic and international students in age and gender adjusted analyses (Odds Ratio or coefficient)

	Unadjusted bivariate comparison†			Age and gender adjusted comparison‡	
Measure	Overall	Domestic	International	International vs. domestic (reference)	95% CI
Depression recognition (%)	83.1	85.9***	74.2***	OR = 0.479***	0.356, 0.643
Mental health problem recognition (%)	94.5	95.6	94.2	OR = 0.706	0.403, 1.236
Help-seeking intention (%)	66.7	64.5***	75.1***	OR = 3.257***	2.145, 4.946
Intended sources of help:					
a. University-based Professional (%)	64.0	56.1***	86.5***	OR = 4.712***	3.242, 6.850
b. Other Professional (%)	73.4	81.2***	51.6***	OR = 0.275***	0.202, 0.374
c. Informal (%)	81.6	83.0*	77.9*	OR = 0.653*	0.452, 0.943
d. Any Professional (university + other, %)	91.9	92.3	90.7	OR = 0.902	0.548, 1.485
Beliefs about usefulness of help (% correct)	73.9	76.3***	66.7***	β=-0.096***	-0.124, -0.067
Would be harmful to deal with problem on their own (% agree)	57.1	61.6***	44.9***	OR = 0.587***	0.462, 0.746
Anti-depressants likely helpful? (% agree)	53.7	57.0***	43.0***	OR = 0.635***	0.498, 0.810
Percent of helpful interventions correctly identified (% correct)	78.1	79.1**	74.6**	β=-0.044**	-0.069, -0.019
Stigma: ‘;weak not sick’ (scale)	0.52	0.35***	1.30***	β = 0.822***	0.717, 0.926
Stigma: ‘dangerous and unpredictable’ (scale)	0.28	0.22***	0.50***	β = 0.214***	0.148, 0.281
Stigma: would you tell anyone if you had a problem like Sammi’s (% agree)	14.7	14.3	15.9	OR = 0.942	0.674, 1.317
Stigma: social distance (scale)	0.62	0.66*	0.52*	β=-0.172*	-0.316, -0.027
Self-reported mental health problem in last year (%)	46.0	51.5***	26.3***	OR = 0.330***	0.254, 0.428
If had a mental health problem, sought help? (% yes)	69.9	71.2*	62.4*	OR = 0.717	0.455, 1.130
If had a mental health problem, reported sources of help used:					
a. Uni-based professional sources (%)	20.0	16.5***	41.2***	OR = 3.152***	1.786, 5.562
b. Other professional sources (%)	81.9	86.3***	50.0***	OR = 0.148***	0.082, 0.268
c. Informal sources (%)	67.0	65.3	76.5	OR = 1.729	0.932, 3.208
If had a mental health problem, would deal with on their own (%)	25.8	24.3*	34.9*	OR = 1.412	0.891, 2.239
Mental health problem among close personal contacts? (% yes)	66.3	76.7***	30.3***	OR = 0.133***	0.099, 0.179
Of those reporting mental health problem in close personal contact, offered help? (% yes)	93.7	93.8	92.9	OR = 0.891	0.296, 2.683
Awareness of confidentiality of university-based health services (% yes)	68.7	71.0	67.6	OR = 0.890	0.744, 1.065
Awareness of confidentiality of community-based health services (% yes)	63.0	79.1***	61.8***	OR = 0.440***	0.367, 0.528

†Bivariate (unadjusted) comparison of international versus domestic students

‡Multivariate (adjusted) comparison of international versus domestic students: Odds ratios (logistic regression) and coefficients (linear regression), adjusted for gender and age

* p < 0.05; ** p < 0.01; *** p < 0.001

*Help-seeking intentions.* The majority of respondents (67%, n = 1,783) indicated that they would seek help if experiencing the problems outlined in the vignette. However, international students were significantly less likely to report that they would seek help compared to domestic students (65% versus 75%), including after adjusting for age and sex (Table 2).

*Intended sources of help.* Of those respondents who said they would seek help (n = 1,193), the majority of students overall would seek help from a university-based professional source, other professional source, or from informal sources (Table 2). At 92%, students overwhelmingly indicated that they would seek help from a professional source (University or other). It is notable that international students were more likely than domestic students to choose university professional sources (86% vs. 56%), and less likely than domestic students to choose other professional sources (52 vs. 81%, Table 2). Finally, international students were less likely than domestic students to choose informal sources of help (78% versus 83%). Most importantly, disregarding *where* professional help would be sought (university versus other), both international and domestic students (91% versus 92%) were highly likely to choose appropriate professional sources of help, which did not differ between student groups (Table 2). International versus domestic student differences in intended sources of help persisted after adjustment for age and sex (Table 2).

*Beliefs about usefulness of help.* For respondents overall, an average of 74% (n = 1,150) of a list of evidence-based helpful sources, including professional and informal, were correctly identified. However, international students identified a significantly lower percentage of helpful sources compared to domestic students (67% versus 76% (Table 2).

Overall, respondents were more likely than not (57%) to correctly identify that it would likely be harmful for Sammi to deal with their problems on their own (Table 2). There was a substantial and significant difference between student groups on this question, with international students less likely to see dealing with problems alone as potentially harmful compared to domestic students (45% versus 62%, Table 2).

More than half of respondents (54%, n = 1,755) correctly identified anti-depressant medication as potentially helpful for Sammi. Again, there was a difference between student groups, with international students significantly less likely to report anti-depressants as helpful compared to domestic students (43% versus 57%).

Of 13 evidence-based helpful interventions, respondents correctly identified an average of 78% (n = 1,688) as helpful (Table 2). International students identified a significantly lower percentage of helpful sources compared to domestic students (75% versus 79%, Table 2).

Differences in beliefs about the usefulness of various forms of help between international versus domestic students remained after adjustment for age and sex (Table 2).

*Personal Stigma*. Scores on the personal stigma measure (range 0–4 with high score = higher stigma) characterising someone with depression as ‘weak, not sick’, were relatively low for the student population as a whole (Mean = 0.52, SD = 1.17, n = 1,406). However, there was a substantial and significant difference between student groups, with international students showing approximately one population standard deviation higher stigma on this measure compared to domestic students (Mean = 1.30 versus 0.35). This was attenuated slightly with adjustment for age and sex, but remained statistically significant.

Similarly, the ‘Dangerous and unpredictable’ personal stigma measure ranges from 0 to 4, with higher values representing higher stigma. By this measure, stigma was relatively low overall (Mean = 0.28, SD = 0.57, n = 1,745). Though the difference was not as large as for ‘Weak, not sick’ (immediately above), international students also showed higher stigma on this measure than domestic students (Mean = 0.50 versus 0.22), including after adjustment for age and sex (Table 2).

For the single-item measure from this scale (that is, ‘would you tell anyone if you have a problem like Sammi’s?’), there was no significant difference between international and domestic students (16% versus 14%, n = 1,755) (Table 2).

*Social distance*. The overall mean score across the full sample was 0.62 (SD = 1.21, n = 1,736) representing a relatively low desire for social distance. In contrast to the above measures of stigma, however, desire for social distance was significantly, though only modestly, lower among international compared to domestic students (Mean = 0.52 versus 0.66, Table 2), including after adjustment for age and sex.

*Self-reported mental health problems.* Nearly half of students (46%, n = 1,768) reported experiencing a mental health problem in the last 12 months (Table 2). Notably, international students were substantially less likely compared to domestic students to report a mental health problem (26% versus 52%, Table 2), including after adjustment for age and sex.

*Experience of help-seeking.* Of those students who reported having a mental health problem (n = 807), a majority (70%) reported having sought help. International students were less likely than domestic students to have sought help for a mental health problem in bivariate analysis (62% versus 71%), but there was no significant difference after adjusting for age and sex (Table 2).

Among those students who reported a mental health problem, respondents were also asked about the sources of help they actually used. Among respondents overall, a majority had sought help from community-based professional sources (82%), followed by informal sources (67%) and University-based professional sources (20%). There was a difference in seeking help from *Other professional sources*, with international students far less likely to seek professional help from outside the University than domestic students (50% versus 86%, Table 2). Differences remained for these two help sources after adjustment for age and sex. Conversely, international students were more likely than domestic students to seek help from university-provided professional sources (41% versus 17%, Table 2). There was no statistically significant difference between the two student groups in seeking help from informal services (Table 2).

Among those who reported that they had a mental health problem, a minority of students overall said that they would ‘try to deal with the problem’ on their own (26%, n = 807). International students were more likely to deal with the problem on their own compared to domestic students in bivariate analysis (35% versus 24%, Table 2), but this difference was not statistically significant after age and sex adjustment.

*Mental health problems among close personal contacts.* Two-thirds (66%, n = 1,458) of the total sample reported that they had a close personal contact who had experienced a mental health problem. International students were far less likely to report mental health problems among close personal contacts than domestic students (30% versus 77%), including after adjustment for age and sex (Table 2).

Of those who had a close personal contact with a mental health problem and replied to whether they had offered help of some kind (n = 664), the vast majority of respondents (94%) indicated that they had offered help. There was no difference between international (93%) and domestic (94%) students with respect to help-offering in this context. The most common form of help, from a closed-ended list of sources provided (Table 3) among combined international and domestic students, was talking to the person and offering support.

**Table 3:** Forms of help offered to close personal contacts who had a mental health problem in the preceding 12 months (n and percent of those reporting offer of help to close personal contacts)

Form of help offered	N	Percent
Talked to the person, offered support	613	90.0
Provided them with self-help materials (e.g., a website or pamphlet)	159	23.3
Suggested they talk to a health professional (e.g., doctor, psychologist)	487	71.3
Assisted them to make an appointment with a health professional	209	30.6
Offered to go with them to see a health professional	273	40.0
Followed up with the person to see if they got professional help	397	58.1

*Confidentiality of health services:* The majority of respondents were aware of the confidentiality of both university- and community-based services, yet 31% and 37% of respondents respectively, were unaware (Table 2, final two rows). There was no difference between international and domestic for awareness of the confidentiality of university-based health services (71% versus 68%). However, international students were less likely than domestic students to report awareness of the confidentiality of community-based health services (62% versus 79%), including after adjustment for age and sex.

## Discussion

This study presents a granular portrayal of mental health-related knowledge, attitudes/stigma, and help-seeking and help-offering behaviours in a university student population, providing information for action at the study site level as well as a contribution to the literature in this area. For students overall, there was some evidence of improved mental health-related knowledge, attitudes and behaviours compared to what has been reported in previous studies (e.g., higher depression recognition and intentions to seek help), but in other areas (e.g., certain measures of stigma), there was little apparent difference over time. With regard to comparisons between international and domestic students, international students most often scored lower on a range of indicators (e.g., depression recognition, awareness of evidence-based forms of help); however, there was also evidence of narrower differences between the two groups than previously reported. Finally, some indicators were more favourable among international students, such as higher help-seeking intentions and lower prevalence of self-reported mental health problems compared to domestic students. Below, findings are compared to previous studies to gauge (1) whether there is evidence of improved mental health-related knowledge, attitudes and behaviours compared to previous studies in students overall, and (2) whether previously observed differences between domestic and international students could be narrowing over time.

Encouragingly, most students correctly identified depression in the vignette (83%), and at a modestly higher level than the ~ 70% that was observed in an earlier study at another Australian university [27] and 75% in a population-based survey of Australians aged 15–25 (including students and non-students) [19]. Further, there was a far narrower gap in recognition rates between domestic and international students in the current study compared to those that have been previously reported. In a 2012 study, for example, international students were 5-times less likely than those born in Australia to recognise depression in a vignette [27].

Another positive finding was the high levels of intentions to seek help if experiencing a problem like those represented in the vignette for both domestic and international students. It is noteworthy that a particularly high proportion of students indicated that they would seek help from a professional source (either through the university or community services). These findings compare favourably to those reported in a 2012 university student study [27], in which only 26% of respondents expressed an intention to seek help from a general practitioner, with only 10% nominating student counselling services. The different preferences for sources of help for domestic (community sources) and international students (university sources) found in the current study may be a reflection of previous access to and familiarity with the Australian healthcare system for domestic students, with the opposite for international students. Cost for community sources may also be a factor. A similar pattern of provider preference was seen in another study of Australian university students [39]. Further, international students might be less likely to use informal sources due to having fewer friends and family nearby.

The willingness of respondents to seek help for a mental health problem was also reflected in responses to the single-item stigma question related to disclosure of a mental health problems, with over 80% of students indicating they would disclose to someone else, and no observed difference between domestic and international students. Unlike in the previously reported study of young people’s stigmatising attitudes, in which participants were likely to rate to a person with depression as unpredictable, in the current study participants were more likely to rate the person as the ‘weak not sick’ than dangerous/unpredictable [20]. A large difference between domestic and international students was observed in the personal stigma measure of ‘weak, not sick,’ which was the largest difference observed on all indicators measured at roughly 1 standard deviation (equivalent to Cohen’s *d ~ 1*), with higher stigma among international students. In addition, international students had higher personal stigma on the ‘dangerous and unpredictable’ scale, suggesting that addressing stigma may be an important target for intervention. This finding must be considered with caution, however, because domestic students showed slightly higher stigma on the social distance measure. Further study is warranted to better understand international versus domestic student differences in stigma.

There was a high proportion of students who correctly identified helpful interventions for mental health problems (~ 74%), which is comparable to a similar measure (77%) used in a decade-old Australian population survey of 15–25 year olds [19]. Other indicators of helpful interventions (e.g., would be harmful to deal with problem on their own, anti-depressants) were comparable to levels among young Australians from a decade ago [19]. International students were consistently lower on four indicators about helpful interventions, suggesting this may be an important intervention priority.

Notably, domestic students were twice as likely as international students to self-report a mental health problem. This is in contrast with other studies suggesting that mental health problems are more prevalent for international students than their domestic counterparts [9]. The 46% of our student sample self-reporting a mental health problem in the last 12 months was higher than a previous WHO multi-country study of university students (including Australia) using validated scale measures which found 38% of students screened positive for at least one 12-month disorder [10]. The closest comparison to our study is provided by a 2018 report on University of Tasmania students, wherein 36% of international and 50% of domestic students reported that “at some point, they had felt the need to seek professional help for one or more of the problems mentioned [mental health, alcohol/substance use, gambling, financial, other] but chose not to do so” [39]. A similar pattern of higher reporting by domestic students was observed, albeit for a broader range of concerns. Interpretation of this disparity in our study is, however, limited. Higher stigma amongst international students could be a barrier to self-report, thus leading to under-reporting; and higher mental health literacy among domestic students would also tend to favour higher self-reporting. Further, while a definition of ‘mental health problem’ was included with the question, students from differing cultural backgrounds may interpret the definition differently, and the nature and severity of these self-reported problems were not queried. Qualitative study would shed further light on this finding as well as elucidating potential differences in willingness to disclose and potentially different meanings ascribed to other key constructs, such as stigma and confidentiality.

Help-seeking behaviour among students who reported experiencing a mental health problem was high (70%) compared to a decade earlier from a survey of ~ 1200 students from three Australian universities, wherein 84% of students experienced psychological distress but only 34% had sought help [40]. Further, there was a far narrower difference between domestic and international students than in previous reports (71% domestic versus 62% international). A 2018 report on a University of Tasmania study found self-reported help-seeking for a mental health problem at 51% among domestic students compared to 17% among internationals [39]. Further, though domestic students were more likely to report having had a close personal contact with a mental health problem in our study, this contrasted with the positive finding that both groups were highly and equally likely (91% for each) to offer forms of help that were consistent with best practice.

Based on concerns raised in a qualitative study of university professional staff [38], we developed questions on student awareness of the confidentiality of health services. The finding that roughly one in four students were reportedly unaware that services were confidential suggests that this could be a barrier to help-seeking that could be addressed through education or awareness campaigns. That international students were less aware than domestic students with regard to the confidentiality of community-based services is likely due to unfamiliarity with and limited utilisation of community services; uncertainty about confidentiality, however, may also constitute a barrier to their use. This finding should be considered in responses to the recent Productivity Commission report that highlighted shortcomings in international student insurance cover for community-based mental health services, with the Commission recommending changes to improve access to and utilisation of public services [9]. On a more positive note, the comparable levels of awareness of the confidentiality of university services between international and domestic students may in part be explained by the university’s efforts to promote the use and confidentiality of university support services.

In summary, this assessment of mental health-related knowledge, attitudes, and behaviours found some important differences between domestic and international students, yet to a lesser extent than anticipated based on previous research [28, 41]. This may be explained by a combination of secular trends (for example, mental health literacy has been steadily improving in the general Australian population [42]), methodological differences between studies, and differences between universities and/or cohorts. It is plausible that the improved levels of mental health-related knowledge, attitudes and behaviours overall and the narrower differences between domestic and international students compared to previous studies are attributable in part to universities’ growing efforts in mental health promotion, psychoeducation and mental health/psychological service provision. This study was motivated primarily by a concern that international students might be faring worse in terms of mental health-related knowledge, attitude, and behaviours as well as mental health [9]; this was to some extent confirmed, though not consistently. Further, it is important to note that there were also areas in which domestic students appear to be faring worse in comparison to international students.

### Limitations and strengths of the study

There is great diversity within the sampled university’s international student population, yet assessments of specific national, regional, or cultural groups was beyond the scope of the present study. In particular, our sample under-represented Chinese students. We also assumed that all respondents interpret the survey questions similarly; however, the same words and phrases may have different meanings for respondents from different backgrounds. Most importantly for the comparisons made in this study, there may be differences by cultural, religious, ethnic, language, nationality or other characteristics. We aimed to mitigate this by using plain language and explicitly defining terms (e.g., mental health problem, confidentiality) wherever possible.

It is also common in voluntary surveys on mental health and wellbeing that respondents with a greater knowledge of or interest in the topic will be more inclined to participate, thus, the sample may over-represent students with personal experience of mental illness, in either themselves or close contacts. This selection bias should have been mitigated, to some extent, by the broad focus of the *Survey* on the student experience and wellbeing (*not* solely mental health and related topics). Social desirability bias may have also influenced self-reports of respondent experiences and behaviours.

A further important limitation of the current study is the low response rate (7%) despite issuing two reminders and the inclusion of prize draws. A University of Tasmania study had a similarly low response rate of 9% [39] and the WHO College Student Initiative Study attained a 7% response rate in Australia, in contrast to the pooled response rate of 46% across the 19 universities in eight participating countries [10]. Accordingly, all findings have to be interpreted with caution until such time as more representative samples can be obtained. Strengths of the study include its census sample frame, the use of established and validated measures where possible which enabled comparison with previous studies, and the close collaboration between the researchers and university professional staff. While these findings generalise, strictly speaking, only to the single large university under study, findings are also likely relevant to other Australian universities.

### Implications for policy & practice

Despite the overall relatively high levels of mental health-related knowledge, attitudes, and behaviours in this study, a number of specific target areas for improvement have been identified in the preceding discussion. For examples, knowledge of best practice, effective interventions for mental health problems, stigma reduction, and the confidentiality of health services in Australia represent specific opportunities for improvement, particularly for international students. Knowledge of university (especially for domestic students) and community (especially for international students) services and supports may also be improved, which may in turn assist in reducing the proportion of students who would deal with a mental health problem on their own. These and other objectives could be addressed through comprehensive mental health and wellbeing strategies, including awareness raising activities and skill development in both students and university staff and the provision of easily accessible supports and services so as to optimise the prevention of illness, the promotion of wellbeing, and early access to effective help for students in distress [12].

Finally, the results of this 2019 survey contribute to the baseline assessment for future evaluation of this university’s Student Mental Health & Wellbeing Strategy. Evaluation of the implementation and effectiveness of specific activities and comprehensive strategies at the university level will be essential to the on-going improvement of efforts to improve university student mental health and wellbeing, both in Australia and internationally [9, 12].

## Electronic supplementary material

Below is the link to the electronic supplementary material.

Supplementary Material 1

## Data Availability

The questionnaires and datasets used in the current study are available from the corresponding author on reasonable request. Anonymized, participant-level data may be made available to external researchers on the basis of a scientifically sound proposal, subject to ethics approval.
