# Supplementary material for: Mental health-related knowledge, attitudes and behaviours in a cross-sectional sample of australian university students: a comparison of domestic and international students
Source: BMC Public Health. 2023 Jan 25;23:170. doi: 10.1186/s12889-023-15123-x (PMC9878763; doi:10.1186/s12889-023-15123-x)
Supplement: Supplementary file 1 — Supplementary Material 1 [file 12889_2023_15123_MOESM1_ESM.docx]

**APPENDICES**

**LaMontagne et al: Mental health-related knowledge, attitudes and behaviours in a cross-sectional sample of Australian university students: A comparison of domestic and international students**

**APPENDIX A**

*Comparison of survey sample demographic characteristics to student population based on university enrolment data.*

|  | | **Student Population %**  **(n = 52,321)** | **Survey sample %**  **(n = 3,743)** |
| --- | --- | --- | --- |
| Gender | Woman  Man | 57*  43* | 68*  29* |
| Disability status | With disability  Not applicable | 7*  93* | 31*  66* |
| Indigenous | Yes  No | 1  99 | 2  98 |
| Socio-economic status | Low  Middle  High | 13  52  34 | 11  47  42 |
| Student type | Domestic  International | 76  24 | 76  24 |
| Age group | Under 21  21 and over | 31*  69* | 24*  76* |
| Campus | Physical #1  Physical #2  Physical #3  Physical #4  On-line/Cloud | 53  9  4  1  23 | 47  18  10  1  23 |

^* Two-sided p < 0.05^

**APPENDIX B**

**Survey question: Depression recognition vignette:**

The next set of questions are about the health problems of a person called ‘Sammi’. Sammi is not a real person, but there are people like Sammi. If you happen to know someone who resembles Sammi in any way, that is a total coincidence.

Sammi is a 21 year old who has been feeling unusually sad and miserable for the last few weeks. Sammi is tired all the time and has trouble sleeping at night. Sammi doesn’t feel like eating and has lost weight. Sammi can’t keep their mind on studies and Sammi’s marks have dropped. Sammi puts off making any decisions and even day-to-day tasks seem too much. Sammi’s parents and friends are very concerned.

**APPENDIX C**

**Survey question on self-reported mental health problems**

A mental health problem is a cluster of symptoms that affects a person’s thinking, emotional state and behaviour, and disrupts the person’s ability to work or carry out other daily activities and engage in satisfying personal relationships. The problem lasts for a period of weeks or more.

In the last 12 months, have you had a **mental health problem**?

Yes 1

No 2

Prefer not to say 3

**APPENDIX D**

**Survey questions on confidentiality of health services**

The use of professional medical services (such as seeing a doctor, counsellor or psychologist) is confidential, meaning that those professionals do not share information about your use of services.

For example, if you used Deakin Counselling and Psychological Services, **no information** about this is provided to your health insurance provider, family, immigration authorities, or employers, and no mention of this would be included in your academic record.

Before today, were you aware that the use **of Deakin’s Medical Services or Deakin’s Counselling and Psychological services are confidential**?

Yes, aware 1

No, not previously aware 2

Wasn’t sure 3

And, before today were you aware that the use of **external community-based professional counselling, medical or psychological services are confidential**?

Yes, aware 1

No, not previously aware 2

Wasn’t sure 3
